# Quantitative assessment of acetabular bone defects: A study of 50 computed tomography data sets

**DOI:** 10.1371/journal.pone.0222511

**Published:** 2019-10-17

**Authors:** Ronja A. Schierjott, Georg Hettich, Heiko Graichen, Volkmar Jansson, Maximilian Rudert, Francesco Traina, Patrick Weber, Thomas M. Grupp

**Affiliations:** 1 Research & Development Department, B.Braun Aesculap AG, Tuttlingen, Germany; 2 Department of Orthopaedic Surgery, Physical Medicine & Rehabilitation, Ludwig-Maximilians-University Munich, Campus Grosshadern, Munich, Germany; 3 Department for Arthroplasty and General Orthopaedic Surgery, Orthopaedic Hospital Lindenlohe, Schwandorf, Germany; 4 Department of Orthopaedic Surgery, König-Ludwig-Haus, Julius-Maximilians-University Würzburg, Würzburg, Germany; 5 Ortopedia-Traumatologia e Chirurgia Protesica e dei Reimpianti d'Anca e di Ginocchio, Istituto Ortopedico Rizzoli di Bologna, Bologna, Italy; 6 Dipartimento di Scienze Biomediche, Odontoiatriche e delle Immagini Morfologiche e Funzionali, Università Degli Studi Di Messina, Messina, Italy; Stanford University, UNITED STATES

## Abstract

**Objectives:**

Acetabular bone defect quantification and classification is still challenging. The objectives of this study were to suggest and define parameters for the quantification of acetabular bone defects, to analyze 50 bone defects and to present the results and correlations between the defined parameters.

**Methods:**

The analysis was based on CT-data of pelvises with acetabular bone defects and their reconstruction via a statistical shape model. Based on this data, bone volume loss and new bone formation were analyzed in four sectors (cranial roof, anterior column, posterior column, and medial wall). In addition, ovality of the acetabulum, lateral center-edge angle, implant migration, and presence of wall defects were analyzed and correlations between the different parameters were assessed.

**Results:**

Bone volume loss was found in all sectors and was multidirectional in most cases. Highest relative bone volume loss was found in the medial wall with median and [25, 75]—percentile values of 72.8 [50.6, 95.0] %. Ovality, given as the length to width ratio of the acetabulum, was 1.3 [1.1, 1.4] with a maximum of 2.0, which indicated an oval shape of the defect acetabulum. Lateral center-edge angle was 30.4° [21.5°, 40.4°], which indicated a wide range of roof coverage in the defect acetabulum. Total implant migration was 25.3 [14.8, 32.7] mm, whereby cranial was the most common direction. 49/50 cases showed a wall defect in at least one sector. It was observed that implant migration in cranial direction was associated with relative bone volume loss in cranial roof (R = 0.74) and ovality (R = 0.67).

**Conclusion:**

Within this study, 50 pelvises with acetabular bone defects were successfully analyzed using six parameters. This could provide the basis for a novel classification concept which would represent a quantitative, objective, unambiguous, and reproducible classification approach for acetabular bone defects.

## Introduction

Revision hip surgery is often associated with acetabular bone defects, which are challenging to quantify. Numerous classification systems have been published in order to categorize those defects [1–3]. However, most of these classification systems are based on plane radiographs and mainly rely on the interpretation of anatomical landmarks, which may lead to poor reliability and repeatability [4–6]. Furthermore, as they are mainly descriptive, it remains difficult to transfer them into pre-clinical testing, implant development, and pre-operative planning. An ideal classification system would provide an objective, unambiguous, surgically relevant and reproducible categorization of bone defects, while being easy to apply. This would ease communication between surgeons, determination of treatment strategy and would also facilitate the prediction and comparison of surgical outcomes.

The application of three-dimensional (3D) imaging such as Computed-tomography (CT) may help to overcome the drawbacks associated with conservative radiograph-based defect classifications [7]. Recently, some approaches towards a more quantitative defect analysis based on 3D-imaging techniques were made, including the analysis of total radial bone loss [7–9], volume loss [7, 10] and remaining bone thickness [7]. Total radial bone loss (TrABL) was among others assessed by Gelaude et al., who conducted the analysis based on segmented CT-data of the defect acetabulum and its anatomic reconstruction [8]. The possibilities to analyze volume loss and remaining bone thickness were mentioned by Horas et al. [7]. Recently, Hettich et al. validated a method to analyze acetabular bone defects in terms of bone volume loss and new bone formation [10]. The study was based on CT-data of defect pelvises and their anatomic reconstructions which were obtained via a statistical shape model (SSM). The bone volumes of defect and native pelvises in four defect sectors were assessed. Bone volume loss and new bone formation were obtained in each sector as absolute value and relative to the native bone volume. These studies pointed out possibilities to analyze acetabular bone defects in a quantitative way. However, these studies using anatomic reconstructions were focused on the analysis of one single parameter (e.g. bone volume loss) and the methods were only applied to a limited number of cases.

In order to evaluate the severity of acetabular bone defects, numerous parameters are of interest, such as bone volume loss, new bone formation, shape of the acetabulum (ovality), support by the cranial roof (lateral center-edge angle, LCE angle), migration of the existing implant, and presence of wall defects. This information could be beneficial in clinical practice for pre-operative planning, prediction and comparison of surgical outcomes, as well as in research and development for implant design and pre-clinical testing.

The objectives of this study were to (1) suggest and define parameters for acetabular bone defect analysis, (2) quantify 50 clinical cases with acetabular bone defects, and (3) present the results and correlations between the parameters.

## Materials and methods

### CT-data

In order to conduct this study, a total of 90 clinical CT-data sets related to acetabular bone defects were kindly provided by the senior hip surgeons HG, VJ, MR, FT and PW from all four involved clinical centers, which was approved by the LMU Munich ethics committee (project no. 18–108 UE). Patients of all age groups with acetabular bone defects of Paprosky type 2A to 3B, including pelvic discontinuity, were selected for this study, whereby CT scans were conducted within pre-operative planning. Patients with existing primary THA, revision THA, infection-related cement spacers, and without implants were included. In accordance with the ethics committee, anonymized CT-data was provided, and patient information was restricted to age and gender. The data sets were screened and the following exclusion criteria were applied to allow successful reconstruction with the SSM [10]: (1) Unilateral scans of the pelvis, (2) data sets with poor image quality, and (3) scans with a distinctive tilted position of the pelvis. After the application of the exclusion criteria, 50 defect hemi-pelvises were included in this study with CT scans conducted between May 2014 and November 2018. 34 were female, 16 male and mean age was 70.02 ± 11.39 (Table 1), average slice thickness and pixel size were 2.37 ± 0.86 mm and 0.79 ± 0.20 mm, respectively.

**Table 1:** Details of the 50 CT-data sets included in the study.

Data set	Age [years]	Gender [F/M]	Clinic	Implant type	Data set	Age [years]	Gender [F/M]	Clinic	Implant type
**01**	62	F	Rizzoli	Muller ring*	**26**	78	F	LMU	Cement spacer
**02**	62	F	Rizzoli	Muller ring*	**27**	49	F	LMU	Cemented PE-cup^¶^
**03**	78	F	Ll	Cage*	**28**	54	M	LMU	Screw cup
**04**	78	M	LMU	Cage*	**29**	47	M	LMU	Cement spacer
**05**	52	F	LMU	Press-fit cup^¶^	**30**	78	F	LMU	No implant
**06**	57	M	Rizzoli	Press-fit cup*	**31**	74	M	LMU	Cement spacer
**07**	65	F	LMU	Cage*	**32**	72	F	LMU	Press-fit cup
**08**	65	F	LMU	Muller ring*	**33**	69	F	LMU	Cement spacer^¶^
**09**	84	F	Rizzoli	Press-fit cup*	**34**	75	F	LMU	Cemented PE-cup
**10**	84	F	Rizzoli	Cemented PE-cup	**35**	69	F	Wrzb	Cage and metal cup*
**11**	55	M	Ll	Muller ring with metal cup*	**36**	79	F	LMU	Press-fit cup
**12**	72	M	Rizzoli	Press-fit cup*	**37**	82	M	Wrzb	Cemented PE-cup^¶^
**13**	76	F	LMU	Cemented PE-cup	**38**	86	F	LMU	No implant
**14**	56	F	Ll	Press-fit cup	**39**	80	F	Wrzb	Press-fit cup
**15**	73	F	LMU	Screw cup	**40**	82	M	Wrzb	Cement spacer
**16**	77	F	Rizzoli	Cemented PE-cup	**41**	75	M	LMU	Screw cup
**17**	56	M	Wrzb	Press-fit cup and plate*	**42**	52	M	LMU	Press-fit cup
**18**	77	F	Wrzb	Screw cup	**43**	75	M	LMU	Press-fit cup
**19**	83	F	LMU	Screw cup	**44**	83	F	LMU	No implant
**20**	59	M	Rizzoli	Press-fit cup^#^	**45**	57	F	LMU	Screw cup^¶^
**21**	57	F	Wrzb	Screw cup	**46**	67	F	Wrzb	No implant
**22**	68	M	LMU	Cement spacer	**47**	76	F	LMU	Cemented PE-cup^¶^
**23**	67	F	Wrzb	Screw cup	**48**	81	F	Wrzb	Press-fit cup*
**24**	68	M	LMU	Cage*	**49**	91	F	LMU	Press-fit cup*
**25**	85	F	LMU	Screw cup	**50**	54	F	LMU	Cage and metal cup*

Clinic names are abbreviated (Ll = Orthopaedic Hospital Lindenlohe; LMU = Orthopaedic Surgery LMU Munich; Rizzoli = Istituto Ortopedico Rizzoli di Bologna; Wrzb = König-Ludwig-Haus Würzburg). Cages and Muller rings were combined with Polyethylene (PE) cups if not otherwise indicated.

*with screws for acetabular component fixation

^¶^with screws for bone fixation

^#^Metal on metal

### Image processing

**Fig 1:**
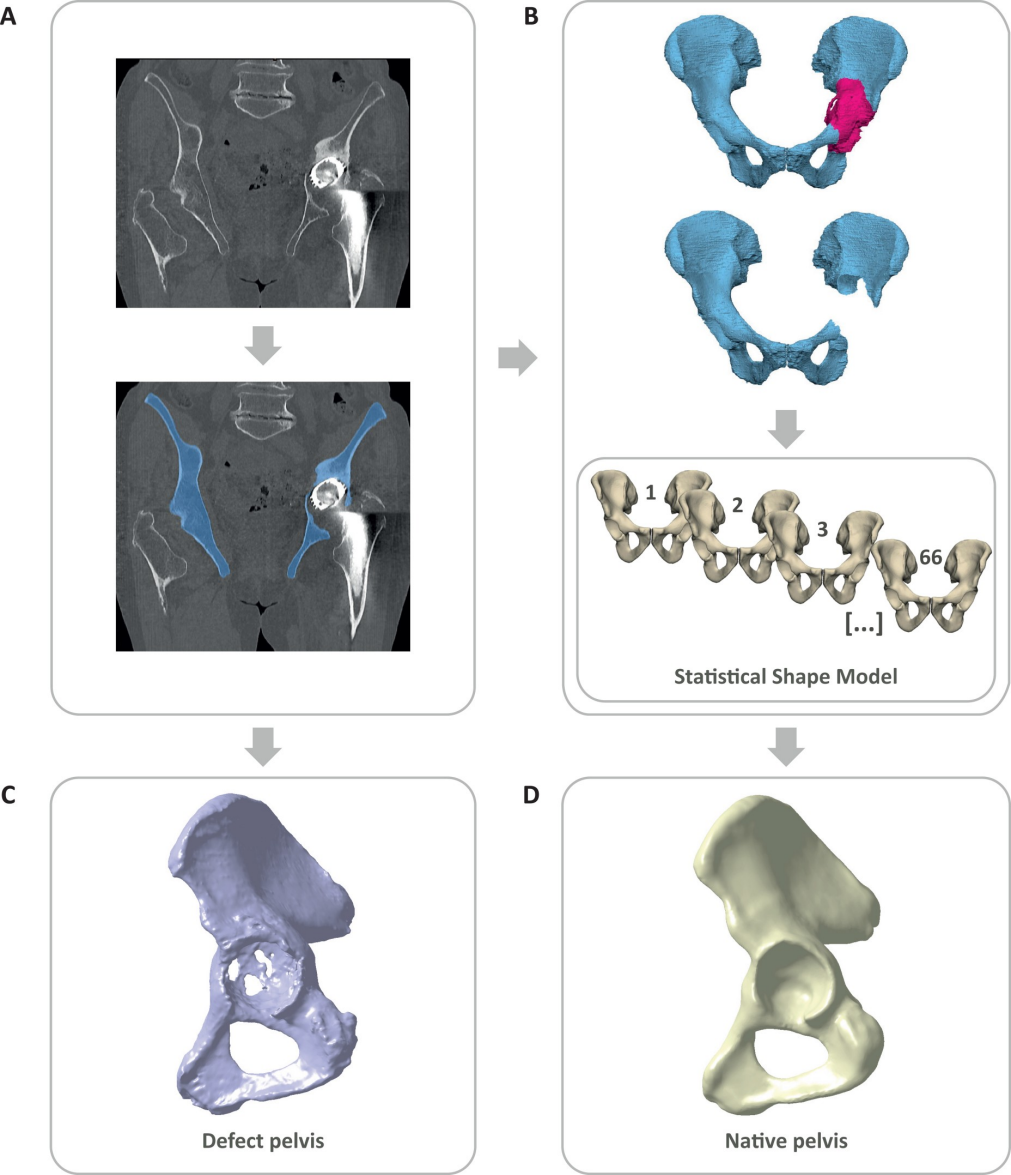
Workflow to obtain defect and native pelvis for the analysis. (A) Segmentation of clinical CT-data set. (B) Masking of the pathological area and application of a statistical shape model to reconstruct the native pelvis. (C) Transformation of CT-data set into solid model of defect pelvis. (D) Transformation of SSM-based reconstruction into solid model of native pelvis.

The analysis was based on solid models of the defect pelvis and the corresponding native pelvis (Fig 1), as previously described and validated [10]. The CT-data set of the defect pelvis was segmented in Mimics 19.0 (Materialise NV, Leuven, Belgium) (Fig 1A). For the reconstruction of the native pelvis corresponding to each defect pelvis, the pathological areas of the segmented CT-data sets were masked and excluded for each defect pelvis individually (Fig 1B). A SSM was trained based on the CT-data sets of 66 healthy pelvises [10]. The SSM is a deformable model that describes the typical 3D shape variation occurring in the training population by so called modes of shape variation that are sorted by their geometric significance. The SSM was fitted only onto the remaining healthy bone structures of each defect pelvis individually by using the first 20 modes of shape variation and the excluded pathological areas could be extrapolated. This resulted in a statistically probable native pelvis model individually for each defect pelvis. After showing promising validation results, comparable to previously applied SSMs, this method was considered to be well suited for the quantitative assessment of acetabular bone defects within the present study. The resulting 3D-models of native pelvis and defect pelvis were exported as Standard-Tessellated-Language (STL) surfaces and mesh correction algorithms were applied in 3-matic (Materialise NV, Leuven, Belgium) and Geomagic Design X (3Dsystems, Rock Hill, USA). In order to process the defect and native pelvis in the CAD-software CATIA V5 (Dassault Systèmes, Vélizy-Villacoublay Cedex, France), both models were transformed into solids using the auto surfacing function in Geomagic Design X (Fig 1C and 1D).

### Quantitative analysis

**Fig 2:**
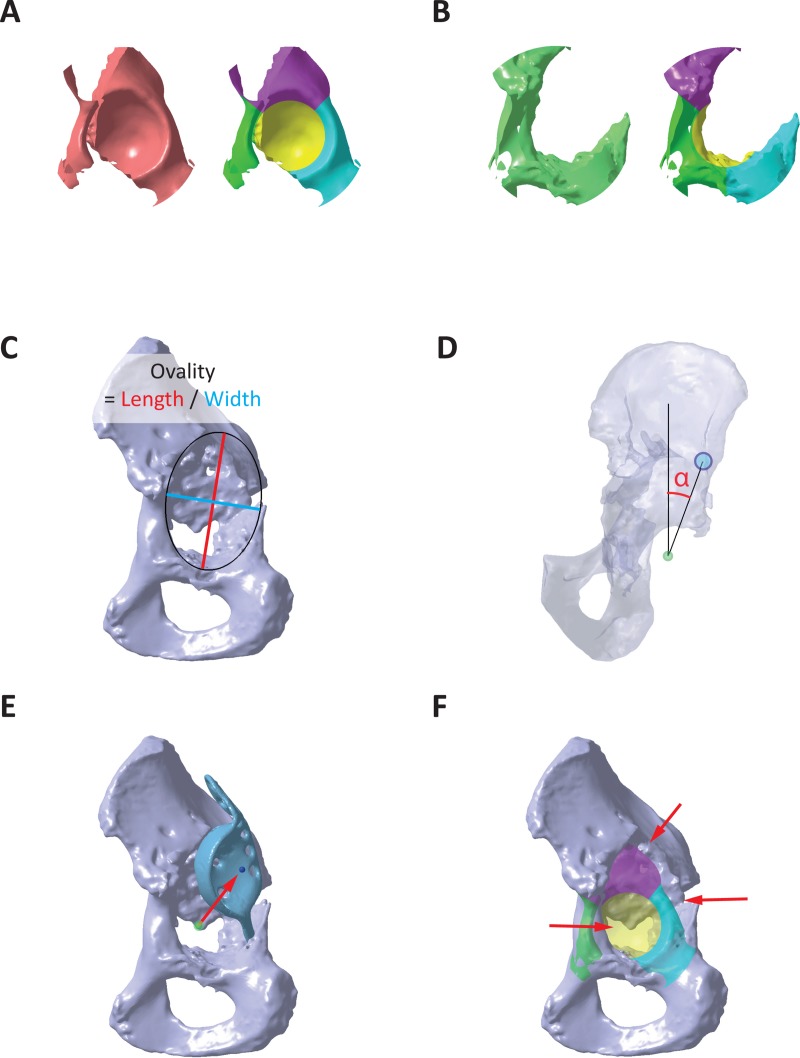
Defect analysis parameters. (A) Bone volume loss in total and in each defect sector calculated using Boolean operations. (B) New bone formation in total and in each defect sector. (C) Ovality of the defect acetabulum given by the ratio of length to width. (D) Lateral center-edge angle defined by the line connecting the most lateral point in the cranial roof with the center of rotation (CoR) and the body Z-axis. (E) Implant migration defined by the distance and direction between the native CoR and the CoR of the existing implant. (F) Wall defect defined by holes and absent bone structures at the acetabular rim (grey = defect pelvis, color transparent = native bone volume in each sector).

The quantitative analysis was performed in CATIA V5 and included six parameters (Fig 2): (1) bone volume loss, (2) new bone formation, (3) ovality of the acetabulum, (4) LCE angle, (5) implant migration, and (6) wall defects. In order to perform a detailed analysis of the defects, four different sectors were defined [10]: Cranial roof (Cranial), anterior column (Anterior), posterior column (Posterior), and medial wall (Medial). To take defects into account, which expand beyond the defined sectors around the acetabulum, bone volume loss in the os ilium, os pubis and os ischii was additionally considered where applicable.

Bone volume loss was analyzed by Boolean operations, whereby the defect pelvis was subtracted from the native pelvis (Fig 2A). New bone formation was also analyzed by Boolean operations, whereby the native pelvis was subtracted from the defect pelvis [10] (Fig 2B). Boolean operations were applied in each defect sector individually. Bone volume loss and new bone formation were calculated as absolute values for each defect sector, and as relative values in relation to the native bone volume in each sector.

Ovality of the acetabulum was measured by fitting an ellipse in the defect acetabulum with the native acetabular plane as basis. Ovality was defined as the ratio of length to width, whereby the ratio 1 represents a circular acetabulum (Fig 2C).

LCE angle was measured based on a technique also used with plane radiographs [11]. The most lateral edge of the cranial roof was marked and a line connecting the native center of rotation (CoR) with the lateral edge was defined. The LCE angle was measured as the angle between the connection line and the body Z-axis, projected on the anterior pelvic plane (Fig 2D).

Implant migration was measured as the difference between the native CoR and the actual implant CoR which was derived by a sphere fitting on the implant surface. Implant migration was analyzed in terms of total distance and distance in medial-lateral, anterior-posterior, and cranial-caudal direction (Fig 2E).

Wall defects were defined as holes with a dimension of 5 mm or larger in length and width or completely absent bone structures at the acetabular rim, indicated by a distance between native and defect rim of 5 mm or larger (Fig 2F).

### Statistical analysis

Statistical analysis included a test for normal distribution of data (Shapiro-Wilk test) and a test for statistical significance between relative bone volume loss in the single sectors (Mann-Whitney-U test). Level of significance was set to p < 0.05. Correlation analysis between the different parameters was performed where applicable. Correlation coefficients were interpreted as moderate correlation (0.5 ≤ R < 0.7), high correlation (0.7 ≤ R < 0.9), and very high correlation (0.9 ≤ R ≤ 1.0) [12].

## Results

**Fig 3:**
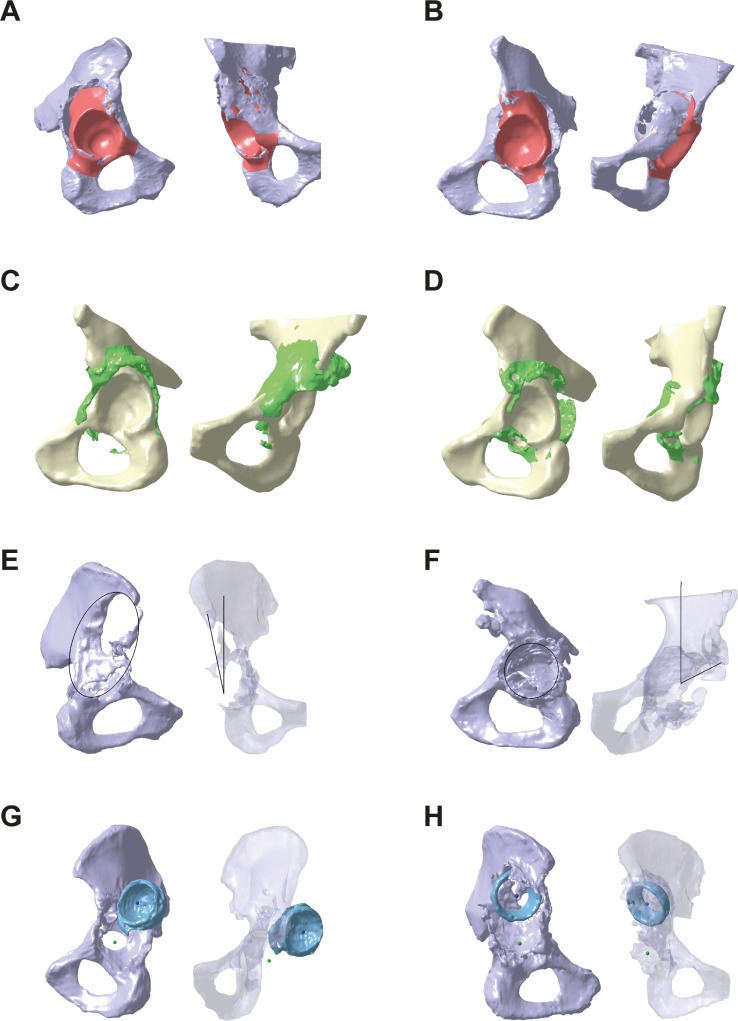
Exemplary defect data sets. (A) Defect pelvis (grey) superimposed with bone volume loss (red) of the case with second-highest relative bone volume loss in Cranial (97.7%). (B) Case with maximum relative bone volume loss in Medial (100.0%). (C) Native pelvis (yellow) superimposed with new bone formation (green) of the case with maximum relative new bone formation in Cranial (31.2%). (D) Case with maximum new bone formation in Posterior (54.1%). (E) Defect pelvis superimposed with ovality and LCE angle of the case with smallest LCE angle (11.6°) and large ovality (1.8). (F) Case with largest LCE angle (63.0°) and smallest ovality (1.0). (G) Defect pelvis superimposed with existing implant (blue) of the case with largest implant migration in lateral direction (39.6 mm). (H) Case with the largest implant migration in cranial direction (52.7 mm).

Bone volume loss, new bone formation, ovality, LCE angle and implant migration in cranial and medial direction are exemplary shown for eight cases (Fig 3).

As most data was found to have a non-normal distribution, the results are presented using the median and [25th, 75th]—percentile values. For better comparison with results reported in literature, mean values ± standard deviations are additionally summarized (Table 2).

**Table 2:** Mean values ± standard deviations for comparison with values given in literature.

	Bone volume loss		Bone formation		Ovality	LCE angle	Implant
							migration
	[ml]	[%]	[ml]	[%]		[°]	[mm]
Cranial	21.2 ± 11.2	47.6 ± 26.3	5.0 ± 3.0	11.2 ± 7.1	1.3 ± 0.2	31.9 ± 11.5	25.1 ± 13.2
Anterior	7.5 ± 3.6	42.6 ± 20.5	3.8 ± 2.8	21.3 ± 15.3			
Posterior	13.8 ± 8.4	41.4 ± 24.9	5.1 ± 3.5	15.5 ± 10.5			
Medial	14.4 ± 5.7	69.2 ± 24.8	2.9 ± 3.4	15.7 ± 18.8			
Total	56.8 ± 21.6	48.7 ± 18.6	16.9 ± 8.1	14.7 ± 7.2			

### Bone volume loss

**Fig 4:**
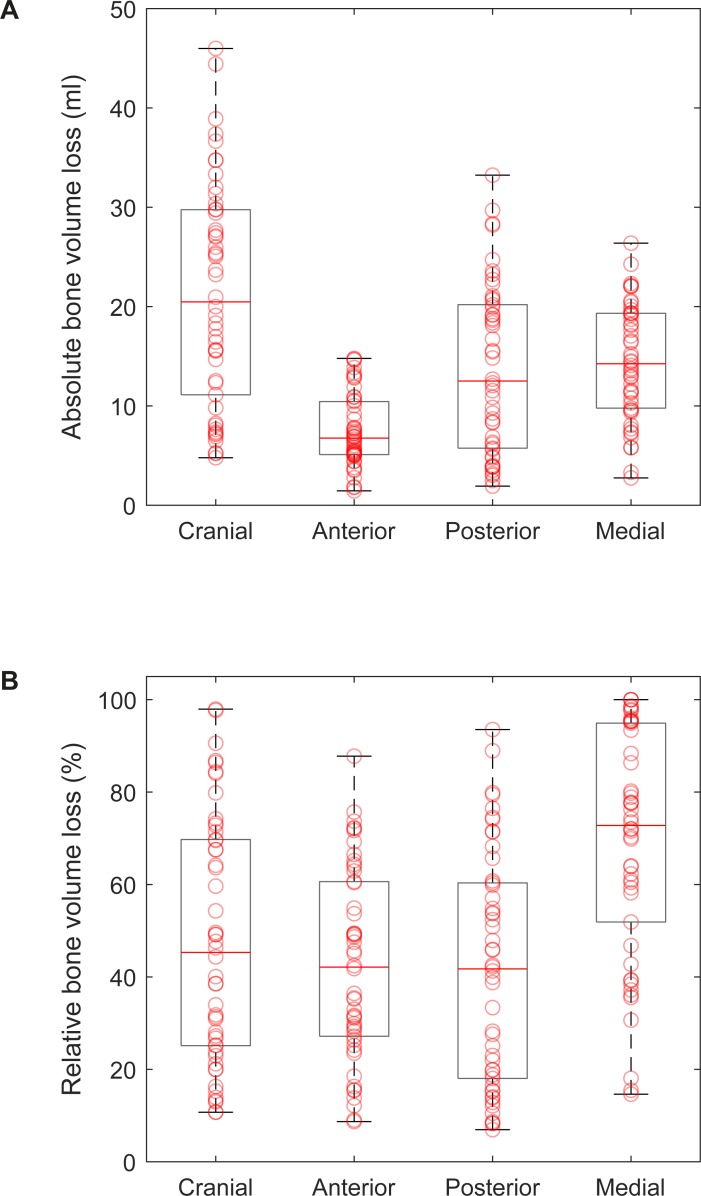
Bone volume loss in each sector. (A) Boxplots of absolute bone volume loss, superimposed with scatter-plots of single values. (B) Boxplots of relative bone volume loss with respect to native bone volume, superimposed with scatter-plots of single values.

Median absolute bone volume loss was 20.5 [10.8, 29.8] ml in Cranial, 6.8 [5.1, 10.5] ml in Anterior, 12.5 [5.7, 20.3] ml in Posterior, and 14.3 [9.7, 19.3] ml in Medial (Fig 4A). Minimum was 4.8 ml in Cranial, 1.5 ml in Anterior, 1.9 ml in Posterior, and 2.8 ml in Medial. Maximum was 46.0 ml in Cranial, 14.8 ml in Anterior, 33.2 ml in Posterior, and 26.4 ml in Medial. In 13/50 cases, bone volume loss in Cranial was accompanied by bone loss in the os ilium and in 4/50 cases, bone loss in Posterior was accompanied by bone loss in the os ischii. In 5/50 cases, pelvic discontinuity was present. Median relative bone volume loss was 45.3 [24.8, 70.2] % in Cranial, 42.1 [27.0, 61.2] % in Anterior, 41.7 [17.7, 60.5] % in Posterior, and 72.8 [50.6, 95.0] % in Medial (Fig 4B). Minimum was 10.7% in Cranial, 8.7% in Anterior, 7.0% in Posterior, and 14.6% in Medial. Maximum was 97.9% in Cranial (case with 97.7% presented in Fig 3A, 97.9% corresponds to case presented in Fig 3E), 87.8% in Anterior, 93.5% in Posterior, and 100.0% in Medial (case presented in Fig 3B). Difference between the groups was significant for Medial and Cranial (p < 0.001), Medial and Anterior (p < 0.001), and Medial and Posterior (p < 0.001). Median and percentile values of absolute bone volume loss were highest in Cranial, whereas median and percentile values of relative bone volume loss were highest in Medial. This difference is related to unequal sizes the defect sectors.

### New bone formation

**Fig 5:**
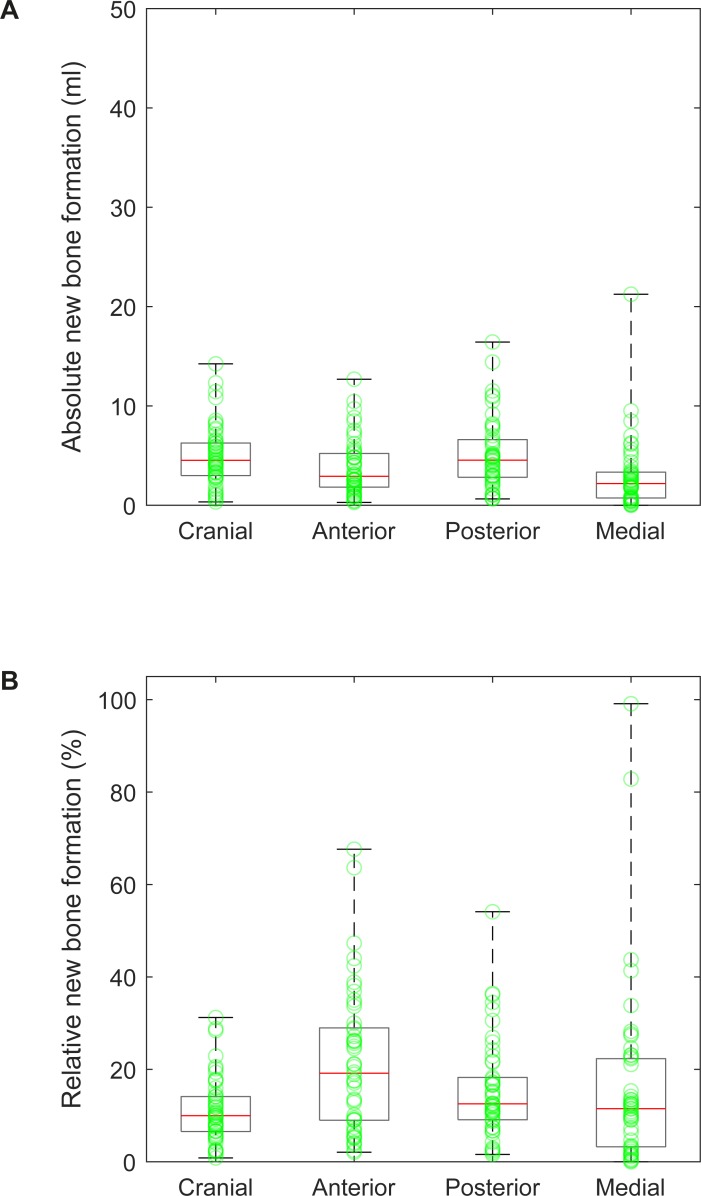
New bone formation in each sector. (A) Boxplots of absolute new bone formation, superimposed with scatter-plots of single values. (B) Boxplots of relative new bone formation with respect to native bone volume, superimposed with scatter-plots of single values.

Median absolute bone formation was 4.5 [3.0, 6.3] ml in Cranial, 2.9 [1.8, 5.3] ml in Anterior, 4.5 [2.8, 6.7] ml in Posterior, and 2.2 [0.7, 3.4] ml in Medial (Fig 5A). Minimum was 0.3 ml in Cranial, 0.3 ml in Anterior, 0.6 ml in Posterior, and 0.0 ml in Medial. Maximum was 14.2 ml in Cranial, 12.7 ml in Anterior, 16.4 ml in Posterior, and 21.3 ml in Medial. Median relative new bone formation was 10.0 [6.4, 14.3] % in Cranial, 19.2 [8.6, 29.2] % in Anterior, 12.5 [9.0, 19.1] % in Posterior, and 11.5 [3.2, 22.4] % in Medial (Fig 5B). Minimum was 0.8% in Cranial, 2.1% in Anterior, 1.6% in Posterior, and 0.0% in Medial. Maximum was 31.2% in Cranial (case presented in Fig 3C), 67.6% in Anterior, 54.1% in Posterior (case presented in Fig 3D) and 99.1% in Medial.

### Ovality of the acetabulum

**Fig 6:**
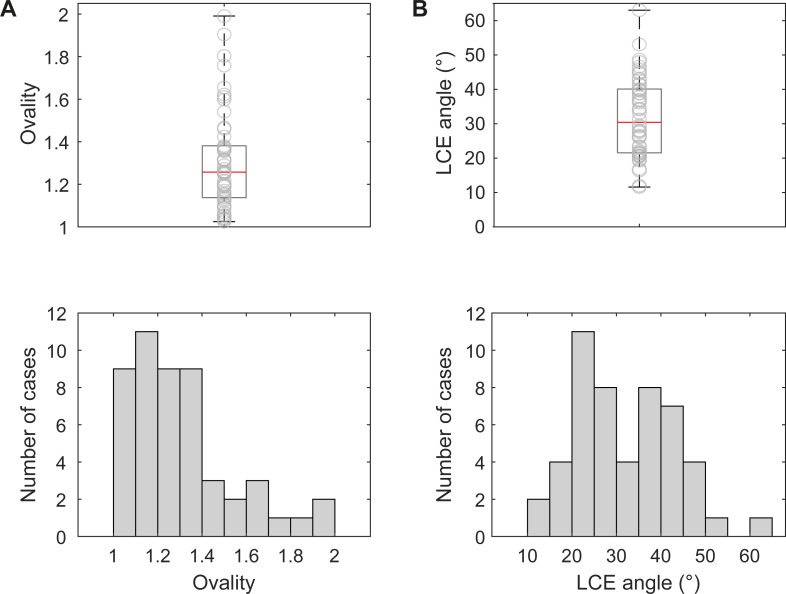
Ovality and lateral center-edge angle (LCE angle). (A) Boxplot of ovality values, superimposed with scatter-plot of single values and histogram of ovality values. Ovality is given as the length to width ratio of the defect acetabulum. (B) Boxplot of LCE angles, superimposed with scatter-plot of single values and histogram of LCE angles.

Ovality was defined as the ratio of length to width of the acetabulum. Median ovality was 1.3 [1.1, 1.4] (Fig 6A). Maximum value was 2.0 and minimum value was 1.0 (case presented in Fig 3F).

### Lateral center-edge angle

Median LCE angle was 30.4° [21.5°, 40.4°] (Fig 6B). Smallest LCE angle was 11.6° was (case presented in Fig 3E), and largest LCE angle was 63.0° (case presented in Fig 3F). In a study about hip dysplasia, LCE angles between 25° and 39° were considered as normal [11]. Applying this to the data of the present study, 18/50 cases showed a normal LCE angle, whereas the remaining cases either showed a reduced (17 cases) or increased angle (15 cases).

### Implant migration

**Fig 7:**
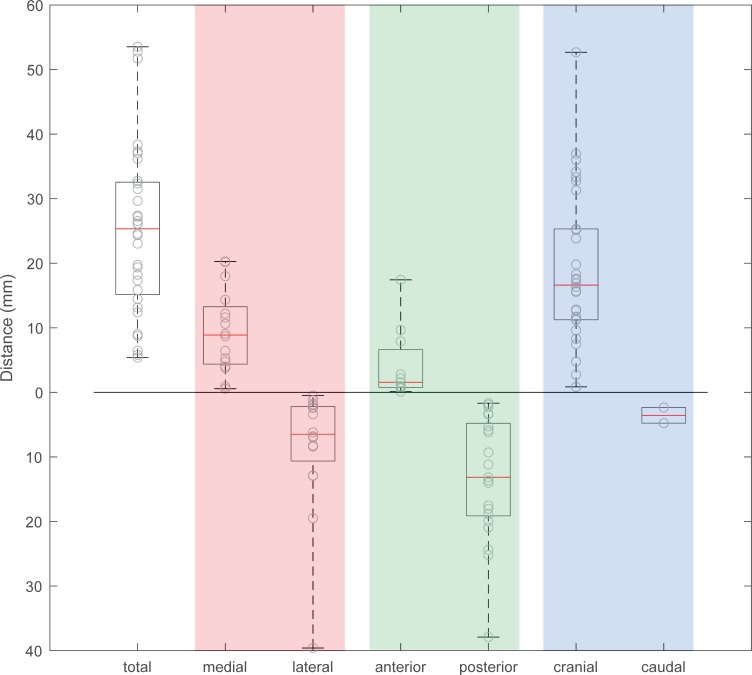
Implant migration. Boxplots superimposed with scatter-plots of single values showing total migration and migration in medial-lateral direction (body X-axis), anterior-posterior direction (body Y-axis), and cranial-caudal direction (body Z-axis).

Implant migration was analyzed in terms of total distance, as well as distance in medial-lateral, anterior-posterior, and cranial-caudal direction (Fig 7). Only cases with segmentable metal implants could be included in this analysis which applied to 32/50 cases. Median total distance was 25.3 [14.8, 32.7] mm with a minimum value of 5.4 mm and a maximum value of 53.5 mm. Median distance in medial direction was 8.9 [4.2, 13.8] mm, in lateral direction 6.5 [2.1, 11.8] mm, in anterior direction 1.6 [0.7, 7.9] mm, in posterior direction 13.2 [4.3, 19.4] mm, in cranial direction 16.6 [11.2, 26.8] mm, and in distal direction 3.6 [N/A, N/A] mm. The maximum distance in medial direction was 20.3 mm, in lateral direction 39.6 mm (case presented in Fig 3G), in anterior direction 17.4 mm, in posterior direction 37.9 mm, in cranial direction 52.7 mm (case presented in Fig 3H), and in distal direction 4.8 mm. 30/32 cases showed a migration in cranial direction. The predominant direction of migration was cranial in 14/32 cases, posterior in 7/32, medial in 7/32 and lateral in 4/32 cases.

### Wall defects

**Fig 8:**
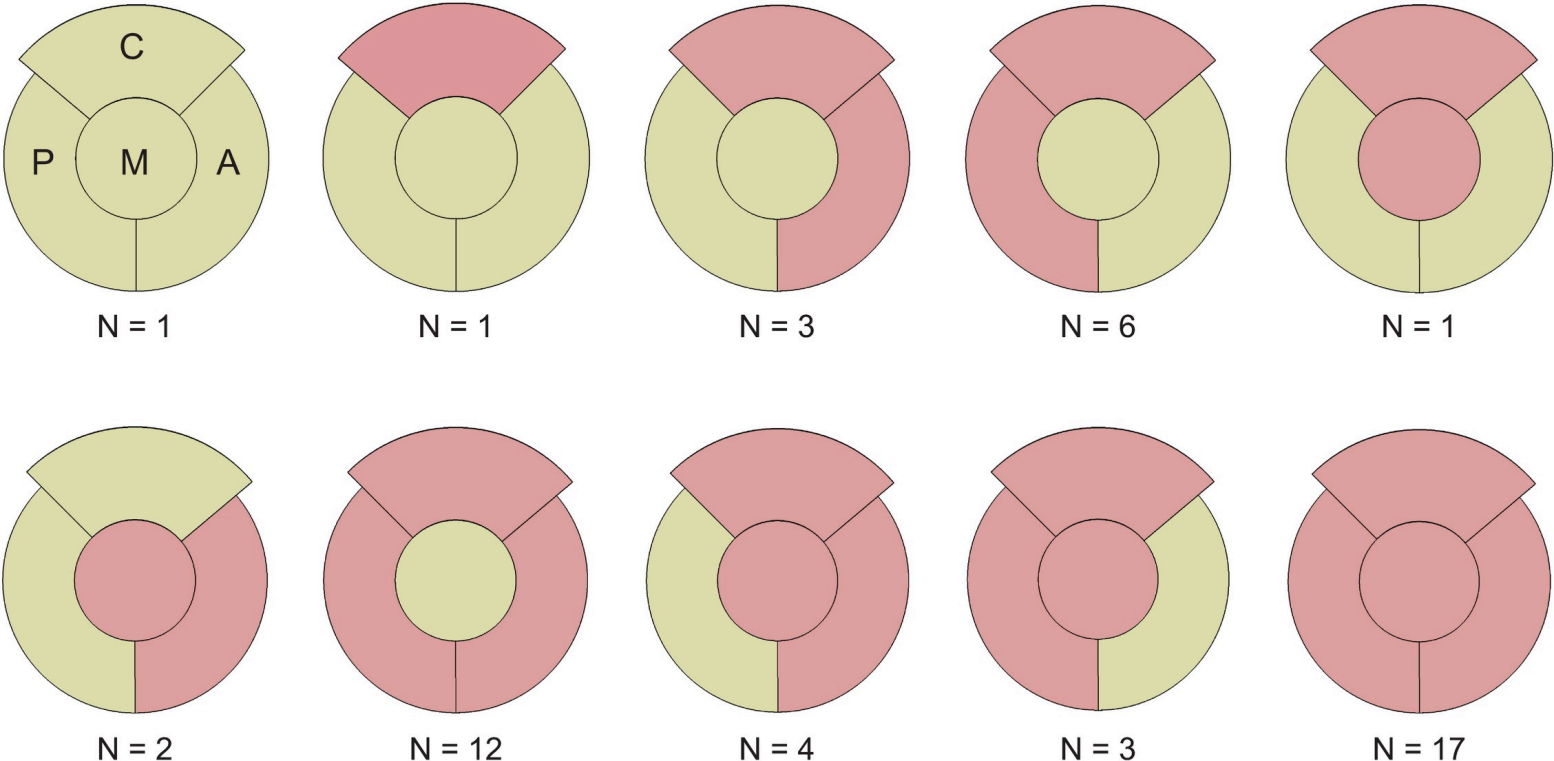
Wall defects. Red color indicates wall defects in the corresponding sectors. Number of concerned cases within this study are indicated below each wall defect combination (C = Cranial, A = Anterior, P = Posterior, M = Medial).

Wall defects, defined as holes with 5 mm or larger in length/width or uncovered rim areas with a distance of 5 mm or more between native and defect pelvis, were present in 49/50 cases (Fig 8). In one case, only Cranial was concerned, whereas the remaining 48 cases showed wall defects in at least two sectors in combination: In three cases, Cranial and Anterior were concerned, in six cases Cranial and Posterior, in one case Cranial and Medial, in two cases Anterior and Medial, in twelve cases Cranial, Anterior and Posterior, in four cases Cranial, Anterior and Medial, in three cases Cranial, Posterior and Medial and in 17 cases, wall defects were present in all four sectors.

### Correlation analysis

Correlation analysis was performed between the different parameters. High correlation was found between total implant migration and migration in cranial-caudal direction (R = 0.89), between implant migration in cranial direction and absolute bone volume loss in Cranial (R = 0.76), and for implant migration in cranial direction and relative bone volume loss in Cranial (R = 0.74). Moderate correlation was found between implant migration in cranial direction and ovality (R = 0.67), for total implant migration and ovality (R = 0.69), for relative bone volume loss in Cranial and ovality (R = 0.64), for relative bone volume loss in Cranial and LCE angle (R = -0.61), and for relative bone volume loss in Cranial and total implant migration (R = 0.65). Moderate correlation was also found for absolute bone volume loss in Cranial and total implant migration (R = 0.66), and for relative bone volume loss in Posterior and implant migration in posterior direction (R = 0.66).

### Derivation of defect groups

Based on relative bone volume loss in each sector, defect groups could be derived. Thereby, thresholds of 15% and 25% were applied to separate clinically relevant bone loss from uncritical bone loss or bone loss caused by measurement inaccuracies. The threshold values were inspired by a study of Gelaude et al. 2011 who defined bone loss greater 15% as slight and greater 25% as moderate [8]. In combination with the assumption that the posterior column is critical for implant stability, a threshold of greater 15% in Posterior and a threshold of greater 25% in Cranial, Anterior and Medial was defined to identify clinically relevant bone loss. Applying these thresholds to the data of the present study, it was found that relevant bone loss was most frequent in Medial (47/50 cases) and Posterior (41/50 cases). Interestingly, relevant bone volume loss in Posterior was in 40/41 cases accompanied by relevant bone volume loss in Medial.

**Fig 9:**
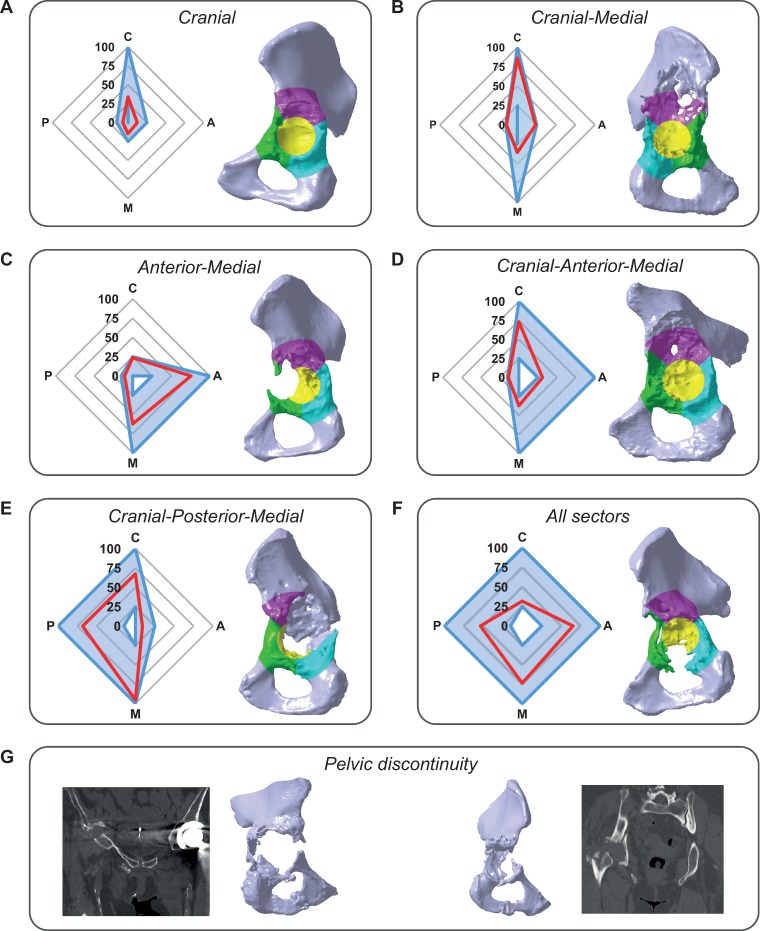
Exemplary application of defect groups. Eight exemplary cases were assigned to the corresponding defect groups. Shown are defect pelvises with defect sectors visualized by different colors and spider plots which show the individual relative bone volume loss (red line) and the corresponding defect group (blue area). Directions in the spider plots are given by Cranial (C), Anterior (A), Posterior (P), and Medial (M). Assignment to *Pelvic discontinuity* was based on 3D-models and CT-images. Group (A) *Cranial* defect. (B) *Cranial-Medial* defect. (C) *Anterior-Medial* defect. (D) *Cranial-Anterior-Medial* defect. (E) *Cranial-Posterior-Medial* defect. (F) *All sectors* defect. (G) *Pelvic discontinuity*.

Based on the relative bone volume loss and the applied thresholds, each clinical case could be assigned to a specific group according to the sectors concerned with relevant bone volume loss (Fig 9). Defect groups are visualized as areas within spider plots (Fig 9). The extent of the areas in the four directions Cranial (C), Anterior (A), Posterior (P), and Medial (M) represents the amount of the corresponding relative bone volume loss. As an example, a case with relative bone volume loss in Cranial of 34.0%, in Anterior of 12.2%, in Posterior of 7.0% and in Medial of 14.6% would be assigned to group *Cranial* (Fig 9A), a case with relative bone volume loss in Cranial of 86.4%, in Anterior of 23.5%, in Posterior of 14.0% and in Medial of 35.5% to group *Cranial-Medial* (Fig 9B), a case with relative bone volume loss in Cranial of 23.7%, in Anterior of 75.7%, in Posterior of 10.5% and in Medial of 62.3% to group *Anterior-Medial* (Fig 9C), a case with relative bone volume loss in Cranial of 73.3%, in Anterior of 31.1%, in Posterior of 13.8% and in Medial of 37.2% to group *Cranial-Anterior-Medial* (Fig 9D), a case with relative bone volume loss in Cranial of 67.5%, in Anterior of 9.0%, in Posterior of 68.3% and in Medial of 95.3% to group *Cranial-Posterior-Medial* (Fig 9E), and a case with relative bone volume loss in Cranial of 31.8%, in Anterior of 65.2%, in Posterior of 53.7% and in Medial of 73.5% to group *All sectors* (Fig 9F). Since a pelvic discontinuity cannot be identified by bone volume loss alone, these cases could be assigned to a separate group based on the CT-images (Fig 9G).

**Fig 10:**
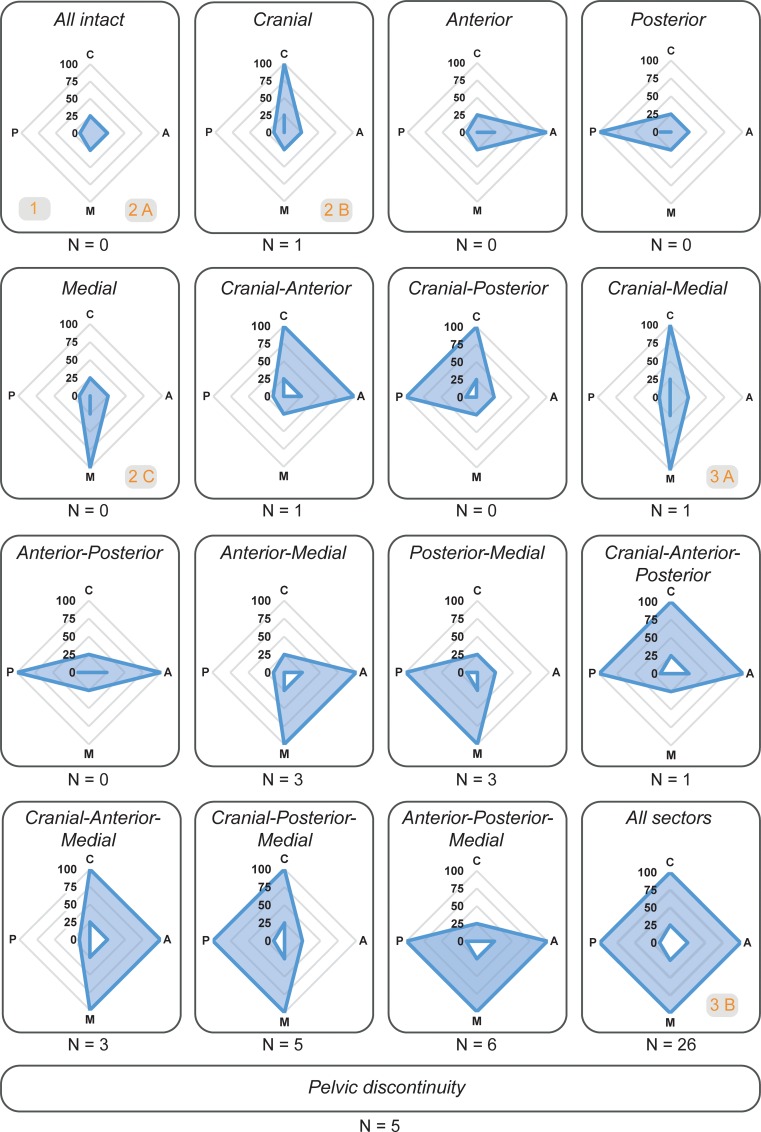
Derived defect groups. Individual cases are assigned to defect groups based on the combination of sectors concerned with relevant bone loss (blue areas). Relative bone volume loss is presented as spider plot with the directions Cranial (C), Anterior (A), Posterior (P), and Medial (M). Number of cases within this study assigned to each defect group is indicated below each plot. Comparison with Paprosky classification is shown in grey boxes in the corresponding group.

Based on the proposed assignment principle, 17 separate defect groups could be derived (Fig 10). Applying this grouping to the data presented in this study, each defect could be unambiguously assigned to a specific defect group. The number of cases within each group is indicated below each spider plot (Fig 10).

## Discussion

The objectives of this study were to (1) suggest and define parameters for acetabular bone defect analysis, (2) quantify 50 clinical cases with acetabular bone defects, and (3) present the results and correlations between the parameters.

Highest relative bone volume loss was found in the medial wall with median and [25, 75]—percentile values of 72.8 [50.6, 95.0] %. Ovality was 1.3 [1.1, 1.4], lateral center-edge angle was 30.4° [21.5°, 40.4°], and total implant migration was 25.3 [14.8, 32.7] mm. Correlation was, besides others, found between implant migration in cranial direction and relative bone volume loss in cranial roof (R = 0.74), as well as ovality (R = 0.67).

Numerous studies have been conducted to quantify bone volume loss of osteolytic lesions in the acetabulum [13–21]. These studies identified a mean bone volume loss between 4.9 ml [13] and 37.9 ml [16], which is lower than the mean bone volume loss observed in the present study (56.8 ± 21.6 ml, Table 2). This difference could be attributed to the fact that the patients included in the previous studies often had a well-functioning total hip arthroplasty (THA). In contrast, the CT data analyzed in the present study was obtained within pre-operative planning and hence often associated with implant migration and large acetabular bone loss. Gelaude et al. published a method to assess the total radial bone loss around the acetabulum [8,9], In a study of 30 acetabular bone defects, they found highest bone loss in the posterosuperior region [9]. In the present study, relative bone volume loss was highest in Cranial and Medial. However, comparison with the results of Gelaude et al. 2013 is limited due to the different sector definitions and different calculation methods for bone loss. In the present study, relative bone volume loss of at least 7.0% was observed in the four sectors of all 50 cases, which indicates multidirectional bone loss. This is in good accordance with previous studies which also found combined defects [8, 22, 23]. Implant migration of more than 5 mm was observed in all 32 analyzed cases in this study. This is in contrast to a recent study of Villatte et al., 2017, who assessed the 5.9-year radiological survival rate of acetabular revision treatments with bone allografts and reinforcement ring in 71 patients and found such high migration rates in only 9 patients [24]. A high correlation between implant migration in cranial direction and relative bone volume loss in Cranial (R = 0.74), as well as a moderate correlation between implant migration in posterior direction and relative bone volume loss in Posterior was found (R = 0.66). Wright and Paprosky already pointed out the influence of migration direction on the involvement of acetabular columns [22], whereby they reported that superior and lateral migration is an indication for a stronger involvement of the posterior column.

Based on the relative bone volume loss in each sector and the application of thresholds, a theoretical total of 17 defect groups could be derived (Fig 10). Comparing the defect groups with the Paprosky classification, group *All intact* would correspond to a Paprosky 1 and 2A defect, group *Cranial* would correspond to Paprosky 2B, group *Medial* would correspond to Paprosky 2C, and group *Cranial-Medial* would correspond to Paprosky 3A. Group *All sectors* would correspond to Paprosky 3B (grey boxes in Fig 10). Hence, the presented quantitative approach could establish a link to the widely used classification system while providing objective, and reproducible information on each defect.

Sandgren et al. 2013 already suggested a quantitative classification system for osteolytic lesions based on the analysis of 206 hips using length measurements [25]. However, since a prerequisite for the application of this method is a clearly defined border around the bone loss, its application to large acetabular bone defects in association with implant migration is limited. The present study quantified acetabular bone defects based on an anatomical reconstruction of the pelvis such that the analysis could also be applied to bone defects without clearly defined borders. Essentially, defect groups could be established based on any of the herein described parameters or based on a combination thereof. The approach based on bone volume loss would enable an unambiguous assignment of defects into groups which is straightforward and unsophisticated. Alternatively, a combination of several parameters could be applied, resulting in a defect characterization matrix. This option would enable a more detailed defect description, which might be helpful to determine treatment strategy, but which would also be more complex and time-consuming to apply.

### Limitations

This study has some limitations. First, the metal implants caused artefacts in the CT-images which may have a detrimental effect on segmentation, and which required manual segmentation under supervision of an experienced radiologist. Second, the assessment of bone volume loss and new bone formation relies on the accuracy of the SSM-based reconstruction of the native pelvis. Reconstruction errors may produce shell-like volumes when subtracting defect pelvis from native pelvis, which are also counted as bone volume loss. This may lead to an over-estimation of bone volume loss. The same applies to the calculation of new bone formation. Third, the proposed defect groups depend on the threshold definition for clinically relevant bone loss. The thresholds of 15% and 25% suggested within this study were defined in consultation with the senior hip revision surgeons HG, VJ, MR, FT and PW and refer to the definitions for slight and moderate bone loss by Gelaude et al. 2011 [8]. These thresholds were used to show the grouping concept and do not represent an exact definition of clinically relevant bone loss. In order to define these threshold values, further studies should be conducted which bring bone loss in relation with treatment strategy and treatment success. Fourth, the proposed defect grouping was based on relative bone volume loss only. However, other clinical parameters were indirectly included due to their correlations with relative bone volume loss and the grouping concept is not restricted to using this parameter. Fifth, it is questionable if 50 cases are enough to capture the whole range of acetabular defects. Nevertheless, to the authors knowledge, there is no study yet about the quantification of acetabular bone defects using several parameters which involves a higher number of cases. Sixth, in order to apply the presented analysis method, a CT scan is required. CT has already been mentioned as promising basis for defect classification [7] and has been widely used during the last years, for example for pre-clinical testing in terms of finite element analysis [26]. This indicates the increasing availability of this imaging technique. Nevertheless, CT is associated with increased irradiation dose and cost in comparison with radiographs as required for previously established classification schemes such as Paprosky [27]. This should be taken into account and the decision whether or not a CT scan is necessary to plan revision surgery should be made carefully and individually for each patient. Seventh, there is no direct clinical consequence in terms of treatment suggestion yet. In order to achieve this, an even larger number of defects should be assessed, also including a follow-up of chosen treatments and treatment success in short- to long-term. Based on this data, defect stages could be defined, and treatment options could be derived thereof.

### Conclusion and outlook

In this study, 50 acetabular bone defects were successfully quantified using six parameters. Based on the results for relative bone volume loss and on the application of thresholds for clinically relevant bone volume loss (Posterior > 15%; Cranial, Anterior, Medial > 25%), defect groups for acetabular bone loss could be derived. This would provide a quantitative, impartial, unambiguous, and reproducible assignment of acetabular bone defects and could also be applied in cases of large bone defects and implant migration.

The quantitative analysis and the assignment to groups could be beneficial in clinical, scientific and engineering applications. In clinical practice, it could ease communication between the surgeons and could provide important information to determine treatment options and to conduct pre-operative planning. Furthermore, using this concept, fully automated assignment of acetabular bone defects to specific defect groups would be possible. In science, it could facilitate the comparison of surgical outcomes due to unambiguous assignment of bone defects. In engineering applications, the data could be used for the development of novel implant concepts and treatment strategies, such as patient-specific implants. Furthermore, it could also enable the transfer of clinically existing defects into pre-clinical in-vitro and in-silico testing, as quantitative data for bone volume loss and defect shape information are now available. Further studies should be conducted in order to enlarge the number of cases to obtain more information on the range of acetabular bone defects and to verify the applied parameters and thresholds for defect grouping. By including the analysis of chosen treatments and treatment success, a link between defect parameters and successful treatment strategies could be established. This would enable the development of a novel quantitative and impartial defect classification which also provides the suggestion of treatment strategies.

## Supporting information

S1 File Summary of the raw data of bone volume for each defect pelvis and corresponding native pelvis. In columns A and B, the case numbers (1 to 50) alongside with the internal identifiers are listed. In columns C to F, the volume of the native pelvis in each sector is listed in mm^3^ for each case. In columns G to J, the volume of the defect pelvis in each sector is listed in mm^3^ for each case. In columns K to N, absolute bone volume loss in each sector in mm^3^ is listed. In columns O to R, relative bone volume loss in each sector (in relation to the native volume in each sector) in % is listed. In columns S to V, absolute new bone formation in each sector in mm^3^ is listed. In columns W to Z, relative new bone formation in each sector (in relation to the native volume in each sector) in % is listed.
(XLSX)

S2 File Summary of the raw data of the analysis of additional parameters (ovality, lateral center-edge (LCE) angle, implant migration, wall defects). In columns A and B, the case numbers (1 to 50) alongside with the internal identifiers are listed. In column C to D, the length and width of the defect acetabulum are listed, which is transferred to a ratio representing the ovality in columns E and F. In column G, the LCE angles are listed. In columns H to N, the total implant migration, migration in medial-lateral, anterior-posterior, and cranial-distal direction in mm are listed. Grey filling indicates that it was not possible to measure implant migration due to the fact that there was no segmentable implant present in the corresponding CT data. In columns O to R, the existence of wall defects in each sector is indicated by “1” in the corresponding column, whereas an intact wall is indicated by “0”.
(XLSX)

S3 File Results of the analysis of bone volume loss as absolute values in ml (sheet 1: Bone_Vol_Loss_Absolute) and as relative values (in relation to *native* bone volume in each sector) in % (sheet 2: Bone_Vol_Loss_Relative). In both sheets, in columns A and B, the case numbers (1 to 50) alongside with the internal identifiers are listed. In sheet 1, columns C to G, the bone volume loss (ml) in each sector, and in total is listed in ml for each case. Below the single measurement values, the median, 25^th^ percentile, 75^th^ percentile, minimum and maximum values are shown, as well as the mean and standard deviations for bone volume loss each sector and for overall bone volume loss. In sheet 2, columns C to F, the bone volume loss (%) in each sector relative to the *native* bone volume in each sector is listed. Below the single measurement values, the median, 25^th^ percentile, 75^th^ percentile, minimum and maximum values are shown, as well as the mean and standard deviations for bone volume loss in each sector. Columns H to R show the defect groups based on the applied thresholds 25% (Cranial, Anterior, Medial) and 15% (Posterior) which were present within the 50 analyzed cases. An “x” indicates the group the corresponding case is assigned to. The number of cases in each group is shown below.
(XLSX)

S4 File Results of the analysis of new bone formation in absolute values in ml (sheet 1: Bone_Form_Abs) and relative values (in relation to the *native* bone volume in each sector) in % (sheet 2: Bone_Form_Rel). ). In both sheets, in columns A and B, the case numbers (1 to 50) alongside with the internal identifiers are listed. In sheet 1, columns C to G, the new bone formation (ml) in each sector and in total is listed in ml for each case. Below the single measurement values, the median, 25^th^ percentile, 75^th^ percentile, minimum and maximum values are shown, as well as the mean and standard deviations for new bone formation in each sector and for overall new bone formation. In sheet 2, columns C to F, the bone volume loss (%) in each sector relative to the *native* bone volume in each sector is listed. Below the single measurement values, the median, 25^th^ percentile, 75^th^ percentile, minimum and maximum values are shown, as well as the mean and standard deviations for new bone formation in each sector.
(XLSX)

S5 File Results of the additional parameter analysis, including ovality (sheet 1), LCE angle (sheet 2), implant migration (sheet 3), and wall defects (sheet 4). In all sheets, in columns A and B, the case numbers (1 to 50) alongside with the internal identifiers are listed. In sheet 1 column C, the ovality for each case is listed. Below the single measurement values, the median, 25^th^ percentile, 75^th^ percentile, minimum and maximum values are shown, as well as the mean and standard deviations. In sheet 2, column C, the LCE angle is listed for each case. Below the single measurement values, the median, 25^th^ percentile, 75^th^ percentile, minimum and maximum values are shown, as well as the mean and standard deviations. In columns F to H, the assignment to the groups according to Wiberg et al. is shown. In sheet 3, implant migration is listed as total distance in mm (column C) and distance in medial-lateral, anterior-posterior, cranial-distal direction (column D to I). Below the single measurement values, the median, 25^th^ percentile, 75^th^ percentile, minimum and maximum values, as well as the mean and standard deviations are shown. In column J, the implant type is listed and in column K to M, the predominant directions based on the values in column D to I are listed. Based on the predominant migration direction, the defect cases could be grouped, as shown in column L and M. In sheet 4, column C to F, the existence of wall defects in each sector is indicated by “1”, whereas an intact wall is indicated by “0”. Based on the combination of concerned sectors, the cases could be grouped (column G). A description of the groups and the number of cases in each group is shown in columns I to K.
(XLSX)

S6 File Summary of the correlation coefficients (R-values) between the single analysis parameters (Variable 1 and Variable 2). Correlations with 0.5 ≤ R < 0.7 are color-coded in green, correlations with 0.7 ≤ R < 0.9 in yellow, and correlations with 0.9 ≤ R in pink, whereby obvious correlations between absolute and relative bone loss, as well as between absolute and relative new bone formation are grayed out.
(XLS)

S7 File Results of the Mann-Whitney test for statistical significance between bone volume loss in the single sectors and new bone formation in the single sectors. Variable 1 and Variable 2 represent the parameters and the table is sub-divided into the four sections absolute bone volume loss, relative bone volume loss, absolute new bone formation, and relative new bone formation. Statistical significance is indicated by a p-value < 0.05 in column D and description “TRUE” in column E (hypothesis).
(XLS)
